# Evidence-based informed consent form for total knee arthroplasty

**DOI:** 10.1186/s13018-023-03647-2

**Published:** 2023-03-02

**Authors:** Satvik N. Pai, Madhan Jeyaraman, Nicola Maffulli, Naveen Jeyaraman, Filippo Migliorini, Ashim Gupta

**Affiliations:** 1 Department of Orthopaedic Surgery, Sri Ramachandra Institute of Higher Education and Research, Chennai, Tamil Nadu 600116 India; 2 Department of Orthopaedics, Faculty of Medicine – Sri Lalithambigai Medical College and Hospital, Dr MGR Educational and Research Institute, Chennai, Tamil Nadu 600095 India; 3 South Texas Orthopedic Research Institute (STORI Inc.), Laredo, TX 78045 USA; 4 Department of Musculoskeletal Disorders, School of Medicine and Surgery, University of Salerno, 84084 Fisciano, Italy; 5 San Giovanni di Dio e Ruggi D’Aragona Hospital “Clinica Orthopedica” Department, Hospital of Salerno, 84124 Salerno, Italy; 6 Centre for Sports and Exercise Medicine, Barts and the London School of Medicine and Dentistry, Queen Mary University of London, London, E1 4DG UK; 7 School of Pharmacy and Bioengineering, Keele University School of Medicine, Stoke-On-Trent, ST5 5BG UK; 8 Department of Orthopaedics, Atlas Hospitals, Tiruchirappalli, Tamil Nadu 620002 India; 9 Department of Orthopaedic, Trauma, and Reconstructive Surgery, RWTH Aachen University Hospital, Pauwelsstraße 30, 52074 Aachen, Germany; 10 BioIntegrate, Lawrenceville, GA 30043 USA; 11 Future Biologics, Lawrenceville, GA 30043 USA

**Keywords:** Informed consent, Total knee arthroplasty, Knee replacement, Medico-legal, Lawsuit, Consent

## Abstract

**Introduction:**

Informed consent documentation is often the first area of interest for lawyers and insurers when a medico-legal malpractice suit is concerned. However, there is a lack of uniformity and standard procedure about obtaining informed consent for total knee arthroplasty (TKA). We developed a solution for this need for a pre-designed, evidence-based informed consent form for patients undergoing TKA.

**Materials and methods:**

We extensively reviewed the literature on the medico-legal aspects of TKA, medico-legal aspects of informed consent, and medico-legal aspects of informed consent in TKA. We then conducted semi-structured interviews with orthopaedic surgeons and patients who had undergone TKA in the previous year. Based on all of the above, we developed an evidence-based informed consent form. The form was then reviewed by a legal expert, and the final version was used for 1 year in actual TKA patients operated at our institution.

**Results:**

Legally sound, evidence-based Informed Consent Form for Total Knee Arthroplasty.

**Conclusion:**

The use of legally sound, evidence-based informed consent for total knee arthroplasty would be beneficial to orthopaedic surgeons and patients alike. It would uphold the rights of the patient, promote open discussion and transparency. In the event of a lawsuit, it would be a vital document in the defence of the surgeon and withstand the scrutiny of lawyers and the judiciary.

## Introduction

Informed consent should protect the rights of the patients and provide them with adequate information before undergoing a medical procedure. Its importance has risen significantly in the past few decades with it being the crux of several lawsuits. It is often the first area of interest for lawyers and insurers when a medico-legal malpractice suit is concerned. Total Knee Arthroplasty (TKA) is one of the most commonly performed orthopaedic procedures worldwide. However, there is a lack of uniformity and standard procedure about obtaining informed consent for the procedure. This leads to informed consent forms which often lack several key aspects and would jeopardise their standing in a court of law. This substantiates the need for a pre-designed, evidence-based informed consent form for total knee replacement cases specifically [1].

## Materials and methods

We extensively reviewed the literature on informed consent in total knee arthroplasty. We also explored literature on the medico-legal aspects of total knee arthroplasty, informed consent and informed consent in TKA. We additionally reviewed the literature on complications occurring after TKA. The electronic databases of PubMed and Cochrane Library were explored using the following search terms and Boolean operators: ‘medico-legal’ OR ‘lawsuit’ OR ‘malpractice’ OR ‘litigation’ AND ‘total knee arthroplasty’ OR ‘knee arthroplasty’ OR ‘knee replacement’ OR ‘total knee replacement’ OR ‘TKA’. The databases were also searched using the terms and Boolean operators: ‘Informed consent’ OR ‘consent’ OR ‘patient consent’ AND ‘total knee replacement’ OR ‘knee replacement’ OR ‘knee arthroplasty. Further searches included the terms and Boolean operators: ‘total knee replacement’ AND ‘complications’ OR ‘adverse events. No restriction in publication date was applied. The manuscript language was restricted to English. In addition, a comprehensive search of reference lists of all identified articles was conducted to identify additional studies. Information about specific medico-legal proceedings involving TKA cases in legal courts, state and national consumer dispute redressal forums, and state medical councils were obtained from different books having a compendium of medico-legal judgements. The results of this literature review were curated, documented and formally published [2].

**Fig. 1:**
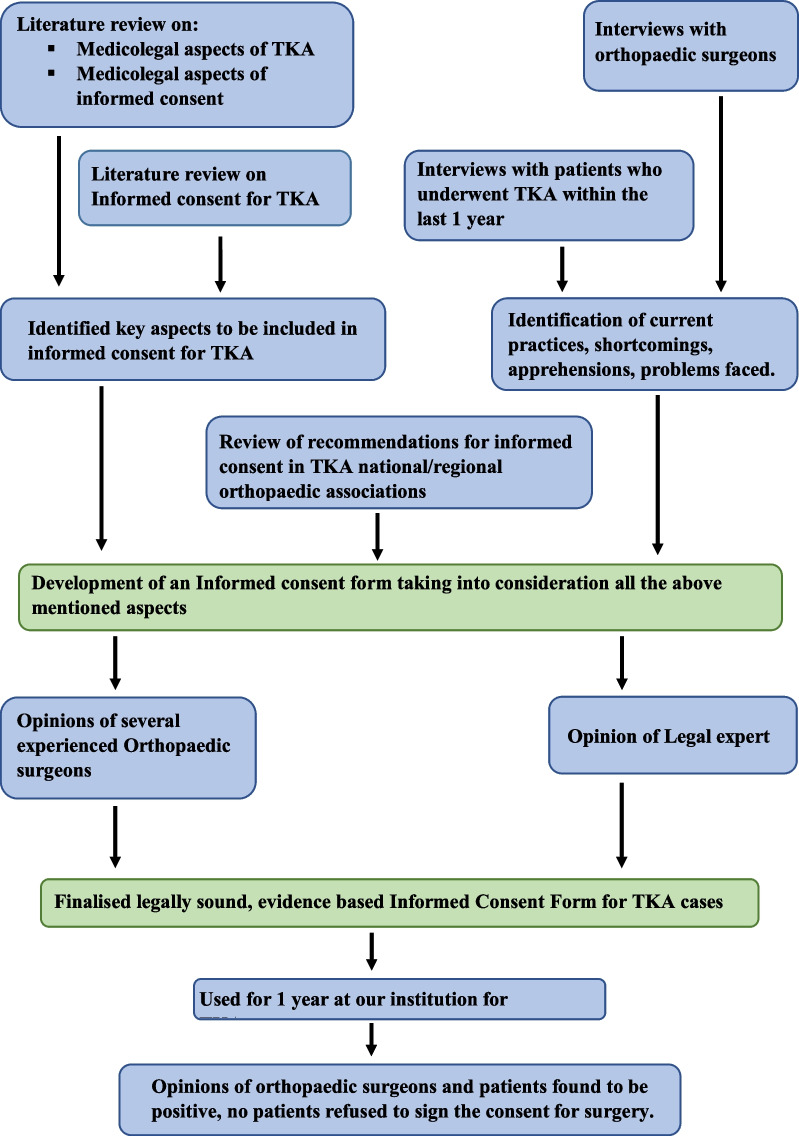
Protocol for drawing the informed consent

We then conducted semi-structured interviews with orthopaedic surgeons from different institutes to understand the common practices about informed consent in TKA, the difficulties they faced, their experiences with contentious informed consent, disputes/concerns patients had raised regarding consent forms and personal experiences in any lawsuits involving TKA cases. We subsequently held semi-structured interviews with patients who had previously undergone TKA in the previous year from the date of the interview. We asked them their personal experience in the process of giving their informed consent, the usefulness of the process, and any doubts which were not satisfactorily addressed in the informed consent. Based on all of the above, we developed an evidence-based informed consent form. This consent form was presented to several experienced orthopaedic surgeons for their personal opinion and suggestions for further improvement. It was also run by a legal expert. Minor modifications were made based on their suggestions, and a final version was prepared and used at our institution for one year. The overall response of orthopaedic surgeons and patients was positive, with no patient refusing to consent for the procedure (Fig. 1).

### Validation of instrument

The prepared tools along with the objectives, blueprints, and criteria rating scale were given to six experts, 3 from the Department of Orthopaedics, Chennai; 1 from the Department of Forensic Medicine, Chennai, 2 from the Department of Community Medicine, Chennai. All the tools were returned after validation of the content.

### Baseline proforma of the study participants

There was 100% agreement in most of the items in the informed consent proforma. The informed consent proforma for study participants had thus 21 items. The modifications were performed as per validators’ suggestions. There was less than 60% agreement on three items of the informed consent proforma and so those items were deleted after the consultation with the expert. Thus, in the present study, the informed consent form had 21 items.

To examine the content validity rate (CVR), the questionnaire was given to 6 experts in the specialities related to the field of the study; the answers were designed based on a three-point Likert scale consisting of necessary, helpful but not necessary, and not necessary. Then, the questionnaire's CVR was assessed; according to the Lawsche table, if the item score was over 0.95, the item was considered as appropriate and necessary. Regarding the obtained scores at this stage, the comments and views of the respondents, and the rethought on the items with lower scores, those that seemed unable to measure the desired concept or those that had a little connection with the issue were excluded.

The indexes of “relevance”, “clarity”, “simplicity” and “ambiguity” were examined. The experts were asked to respond to two questions: (1) the viewpoints they believed should be imposed and (2) suggestions for the items that should be entered into the questionnaire. A separate content validity index (CVI) was calculated for each item and scale. Thus, we calculated scale-content validity index S-CVI/Average for the overall six constructs (1.00 + 1.00 + 0.83 + 1.00 + 1.00 + 0.83)/6 = 0.94 [3].

## Results

**Fig. 2:**
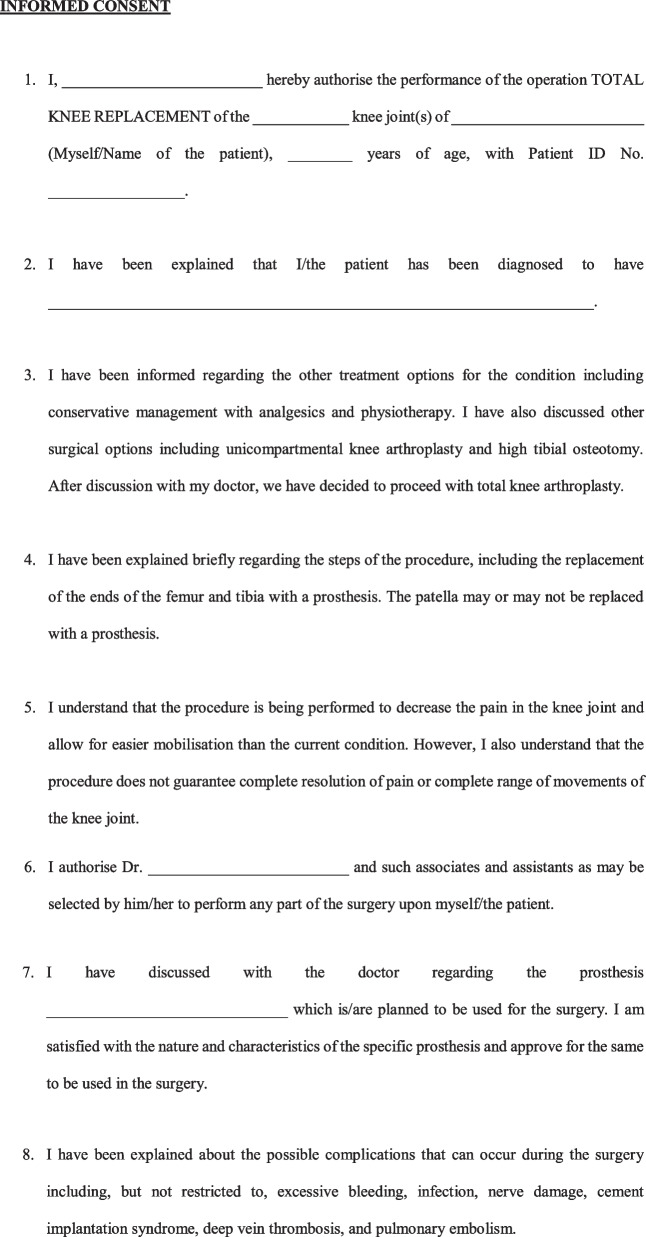

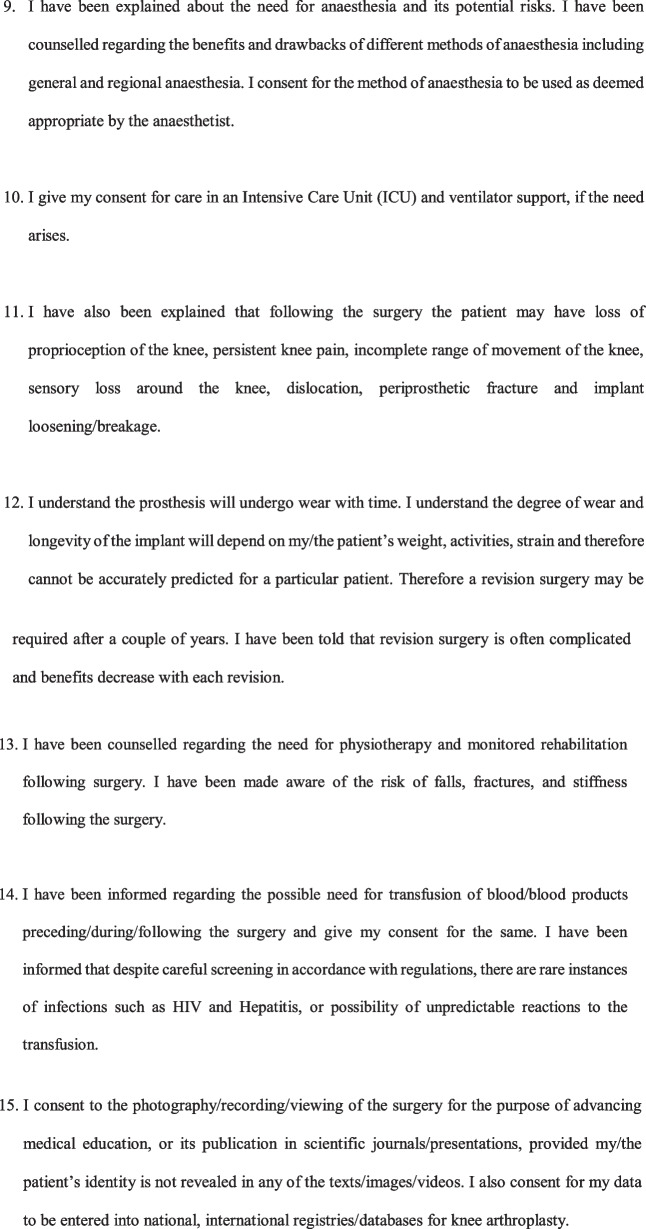

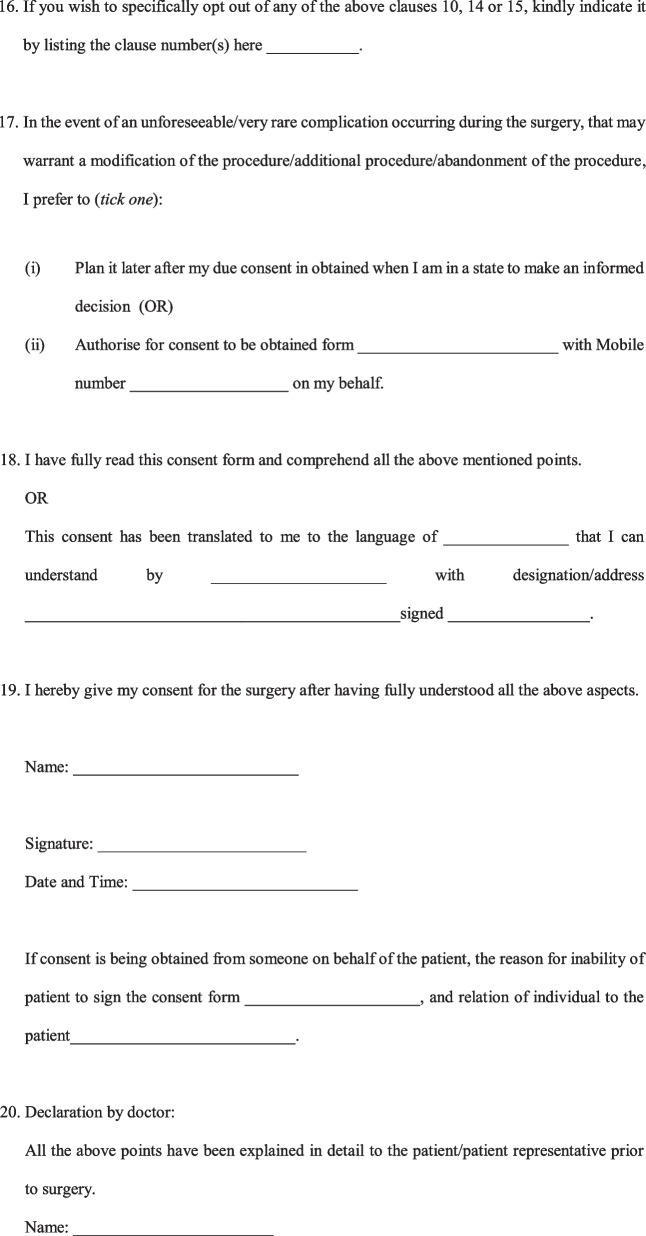

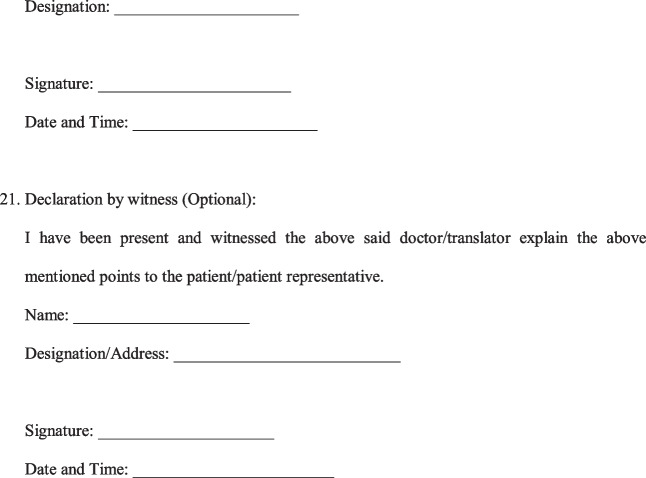
Evidence-based informed consent form

A Legally sound, Evidence-based Informed Consent Form for Total Knee Arthroplasty (Fig. 2) was developed.

## Discussion

Informed consent is often the most vital document in the defence of a malpractice lawsuit. However, almost all available literature suggest that informed consent taken for TKA is incomplete and requires improvement. These shortcomings in informed consent leave orthopaedic surgeons liable to malpractice claims, and can be used against them in a court of law. The use of generic forms with blank spaces for TKA cases has been found to lead to insufficient documentation of clinically significant complications, and hence the use of standardised, procedure-specific consent forms for TKA has been recommended [4]. It is important to document the diagnosis and discuss alternative treatment options with patients before surgery. Alternative treatment options are one of the most commonly missed parts of informed consent forms [5]. Patients should receive a full explaination about the procedure in brief in a way that patients can understand. It is also vital that the surgeons discuss the goal of the procedure so that patients have realistic expectations from the surgery. The expected outcome and activities which they will be able to undertake (or not) after TKA was a very important part of the informed consent in the view of patients [6]. Clarification that the surgery does not guarantee complete resolution of pain is paramount. This discussion with the patient on the expected outcome of TKAhas been found to decrease medical malpractice claims [7]. A discussion on the prosthesis that is planned to be used is another aspect that is often wrongly omitted. Orthopaedic surgeons must discuss with the patient regarding the implant options available, the implant the surgeon feels most appropriate for the patient and the reasons for this, the scientific evidence regarding the implant, and also disclose any financial relationships or conflicts of interest for the surgeon [8]. This is extremely important, as, in the event of implant failure/breakage, patients often allege the use of poor-quality implants [9]. Patients should also be informed about the longevity of the prosthesis and the possibility of the need for a revision procedure [6].

Patients should be explained about the common complications occurring after TKA, and some of the less common but serious complications. While most consent forms list non-specific complications such as infection, bleeding, neurovascular injury, most consent forms do not address complications specific to TKA such as knee stiffness, prosthesis wear/loosening, or persistence of pain [4]. There remains confusion regarding which complications to list in the informed consent, and it is advisable to follow the guidelines/recommendation of a national/regional association of orthopaedic surgeons for the same. Such a recommendation is however not available in most countries, and a major reason for a lack of uniformity and absence of standard practice. Our consent form lists all the relevant complications based on the recommendation by the British Orthopaedic Association [10] as well as taking into account the common causes of litigation in TKA cases [2, 11]. Another often neglected aspect is counselling by orthopaedic surgeons regarding the need and course of post-operative physiotherapy and rehabilitation. Though some may contend that this is not essential as it is not a part of the surgical procedure, post-operative physiotherapy and rehabilitation should be explicitly mentioned, as their implication is realised only in the event of a fall during the post-operative period or an adverse outcome occurring as a result of the patient not following the surgeon’s advice with regards to rehabilitation [2, 9]. Obtaining prior consent for photography/recording of the surgery for education purposes/publication in scientific journals is always recommended as a part of research ethics.

In the event of an unexpected occurrence during the operation, warranting a modification/abandonment of the procedure, with patients unable to consent being under the effect of general anaesthesia/sedation, we recommended taking in writing the preference of the patient to either plan the procedure at a later date or have a designated representative to consent for any modification/additional procedure should need arise. This would not only safeguard the surgeon who takes actions in the best interest of the patient in the event of an unforeseeable complication, but also uphold the interests of the patient to avoid additional surgery on account of being unable to give consent at the time. The language of the consent form poses a challenge when dealing with individuals who cannot read English language. For the consent to be valid even for such patients in a court of law, we recommend documenting the language it was translated to, the details of the translator and the signature of the translator as well. The translator can be any individual who can read English and translate it to a language the patient understands. Obtaining the signature of the patient and doctor obtaining the consent is paramount. The signature of a witness is not required by law, but is recommended as an additional safeguard measure. This form is currently available only in English, and it is desirable that cross cultural validations are undertaken so that it can be used in other languages [12, 13].

## Conclusion

The use of legally sound, evidence-based informed consent for total knee arthroplasty cases would be beneficial to orthopaedic surgeons and patients alike. It would uphold the rights of the patient, promote open discussion and transparency. In the event of a lawsuit, it would be a vital document in the defence of the surgeon and withstand the scrutiny of lawyers and the judiciary.

## Data Availability

All the data is contained within this manuscript.
