# Correction: Evidence-based informed consent form for total knee arthroplasty

**DOI:** 10.1186/s13018-024-04603-4

**Published:** 2024-02-12

**Authors:** Satvik N. Pai, Madhan Jeyaraman, Nicola Maffulli, Naveen Jeyaraman, Filippo Migliorini, Ashim Gupta

**Affiliations:** 1 Department of Orthopaedic Surgery, Sri Ramachandra Institute of Higher Education and Research, Chennai, Tamil Nadu 600116 India; 2 Department of Orthopaedics, Faculty of Medicine – Sri Lalithambigai Medical College and Hospital, Dr MGR Educational and Research Institute, Chennai, Tamil Nadu 600095 India; 3 South Texas Orthopedic Research Institute (STORI Inc.), Laredo, TX 78045 USA; 4 Department of Musculoskeletal Disorders, School of Medicine and Surgery, University of Salerno, 84084 Fisciano, Italy; 5 San Giovanni di Dio e Ruggi D’Aragona Hospital “Clinica Orthopedica” Department, Hospital of Salerno, 84124 Salerno, Italy; 6 Centre for Sports and Exercise Medicine, Barts and the London School of Medicine and Dentistry, Queen Mary University of London, London, E1 4DG UK; 7 School of Pharmacy and Bioengineering, Keele University School of Medicine, Stoke-On-Trent, ST5 5BG UK; 8 Department of Orthopaedics, Atlas Hospitals, Tiruchirappalli, Tamil Nadu 620002 India; 9 Department of Orthopaedic, Trauma, and Reconstructive Surgery, RWTH Aachen University Hospital, Pauwelsstraße 30, 52074 Aachen, Germany; 10 BioIntegrate, Lawrenceville, GA 30043 USA; 11 Future Biologics, Lawrenceville, GA 30043 USA

**Correction: Journal of Orthopaedic Surgery and Research** 10.1186/s13018-023-03647-2

Following publication of the original article [1], the authors identified an error in the author name of Nicola Maffulli.

The incorrect author name is: Nicola Maffuli

The correct author name is: Nicola Maffulli

The author group has been updated above and the original article [1] has been corrected.
